# Urinary exosomal miRNA-663a shows variable expression in diabetic kidney disease patients with or without proteinuria

**DOI:** 10.1038/s41598-022-26558-4

**Published:** 2023-03-18

**Authors:** Nisha Sinha, Veena Puri, Vivek Kumar, Ritambhra Nada, Ashu Rastogi, Vivekanand Jha, Sanjeev Puri

**Affiliations:** 1 Centre for Stem Cell Tissue Engineering and Biomedical Excellence, Panjab University, Chandigarh, India; 2 Centre for Systems Biology and Bioinformatics, Panjab University, Chandigarh, India; 3 Department of Nephrology, Post Graduate Institute of Medical Education and Research, Chandigarh, India; 4 Department of Histopathology, Post Graduate Institute of Medical Education and Research, Chandigarh, India; 5 Department of Endocrinology and Metabolism, Post Graduate Institute of Medical Education and Research, Chandigarh, India; 6 The George Institute for Global Health, New Delhi, India; 7 Department of Biotechnology, University Institute of Engineering and Technology (UIET), Panjab University, Chandigarh, India

**Keywords:** Computational biology and bioinformatics, Nephrology

## Abstract

Heterogeneity in the Diabetic Kidney Disease (DKD) diagnosis makes its rational therapeutics challenging. Although albuminuria characterizes DKD, reports also indicate its prevalence among non-proteinuric. Recent understanding of disease progression has thus inclined the focus on proximal tubular cell damage besides the glomeruli. A non-invasive approach exploiting exosomal miRNA derived from human kidney proximal tubular cell line was, hence, targeted. Upon miRNA profiling, three miRNAs, namely, hsa-miR-155-5p, hsa-miR-28-3p, and hsa-miR-425-5p were found to be significantly upregulated, while hsa-miR-663a was downregulated under diabetic conditions. Among these, hsa-miR-663a downregulation was more pronounced in non-proteinuric than proteinuric DKD subjects and was thus selected for the bioinformatics study. Ingenuity Pathway Analysis (IPA) narrowed on to IL-8 signaling and inflammatory response as the most enriched ‘canonical pathway’ and ‘disease pathway’ respectively, during DKD. Further, the putative gene network generated from these enriched pathways revealed experimentally induced diabetes, renal tubular injury, and decreased levels of albumin as part of mapping under ‘disease and function’. Genes target predictions and annotations by IPA reiterated miR-663a’s role in the pathogenesis of DKD following tubular injury. Overall, the observations might offer an indirect reflection of the underlying mechanism between patients who develop proteinuria and non-proteinuria.

## Introduction

The frequency of type 2 diabetes mellitus (T2DM) is rising at an alarming rate. It is predicted by the Institute for Alternative Futures, that the number of diabetics will increase by 54% between 2015 and 2030^1^. Almost one-third of type 2 diabetics end up suffering from diabetic kidney disease (DKD) which is characterized by progressively increasing proteinuria followed by a gradual decline in glomerular filtration rate (GFR) and is often detectable in the advanced stages of DKD. A proportion of patients also show GFR decline without proteinuria, the so-called non-proteinuric DKD (NPDKD)^2^. In an Italian multicentric Renal Insufficiency And Cardiovascular Events study, 56.6% of all the Type 2 diabetics with renal impairment were normoalbuminuric, 30.8% were moderately albuminuric and 12.6% were severely increased albuminuric^3^. Moreover, possible effects of other factors including cholesterol emboli, interstitial fibrosis, etc. in renal function loss cannot be ruled out. Further, at times, kidney function deterioration precedes proteinuria^4^. Hence, a need exists for the identification of biomarkers reflecting the early effects of disease during the development and progression.

MicroRNAs (miRNAs) are non-coding RNAs that translationally repress or degrade RNA by binding to the 3’ untranslated region of the mRNA. Their presence in the urine makes them an ideal candidate for diagnosing DKD early^5^. There are several kinds of extracellular vesicles (EVs) in urine, with exosomes being the best characterized^6^. Exosomes are 20–100 nm-sized vesicles formed by the fusion of multivesicular bodies with the plasma membrane and carry protein and/or nucleic acid cargo of renal dysfunction or structural injury^7^. Since exosomal miRNAs are protected from endogenous RNase, their stability is superior to free urinary miRNAs^6^. Moreover, according to Kamal et al. exosomal miRNAs are most commonly used for biomarker studies over non-exosomal miRNAs^8^. Growing evidence suggested the importance of urinary exosomes (UEs) in DKD pathogenesis^9–11^. Though these have been explored as a biomarker for DKD, a puzzle still exists to differentiate non-proteinuric from proteinuric DKD. Nowak et al. study showed that the progression of DKD in the absence of proteinuria was associated with renal tubular injury, as seen in the cases of non-proteinuric patients who have more significant tubulointerstitial fibrosis and atrophy than patients with proteinuria. His study also concluded that the proximal tubular injury developed early in DKD and contributed to the progression of the disease^12^.

In this study, therefore, we first identified the dysregulated miRNAs from proximal tubular cells (PTC)-derived exosomes involved in DKD by miRNA profiling. The targeted miRNAs were then validated under in vitro conditions followed by their expression analysis in the UEs of DKD subjects with varying degrees of albuminuria. The miR-663a was the only miRNA to be differentially expressed between non-proteinuric and proteinuric DKD. The target genes retrieved by various bioinformatics tools for enriched canonical pathways were analyzed by Ingenuity Pathway Analysis (IPA) software. The genes from these pathways were constructed into a topological network, which when overlaid with the ‘diseases and functions’ module revealed endocrine system disorders, renal tubular injury, and decreased levels of albumin as relevant functions during DKD. Overall, these findings identified miR-663a as a novel molecule involved in segregating proteinuric from non-proteinuric DKD.

## Methods

### Study definitions, setting, and subjects

Patients attending the outpatient clinics in the Department of Nephrology and Endocrinology at the Postgraduate Institute of Medical Education and Research (PGIMER), Chandigarh, India were screened for enrollment after due approval from PGIMER (PGIMER; PGI/IEC/2014/517).  All the methods and the experiments were carried out in accordance with relevant guidelines and regulations. T2DM was defined as the diagnosis of Type 2 diabetes mellitus as per the prevailing American Diabetes Association (ADA) definition. Chronic kidney disease (CKD) was defined as either CKD Epidemiology Collaboration (CKD-EPI) estimated GFR (eGFR) < 60 ml/min/1.73m^2^ or 24-h urine protein excretion ≥ 150 mg for at least 3 months. In patients with T2DM and CKD, diagnosis of DKD was based on the attending clinician’s judgment and exclusion of non-diabetic causes of CKD. Proteinuric DKD in patients with diabetes mellitus and CKD was defined as CKD-EPI eGFR < 60 ml/min/1.73m^2^ and 24-h urine protein excretion of ≥ 500 mg. Non-proteinuric DKD in patients with diabetes mellitus and CKD was defined as CKD-EPI eGFR < 60 ml/min/1.73m^2^ and 24-h urine protein excretion of < 500 mg. Prospective living kidney donors were defined as healthy if they had eGFR ≥ 60 ml/min/1.73m^2^, 24-h urine protein excretion < 150 mg, and no co-morbidities based on pre-transplant screening protocol. This protocol included clinical details, blood, and urine investigations, and imaging of the kidneys and urinary tract. Written informed consent was obtained from the recruited patients and healthy volunteers after explaining the details about the aim of the study, the extent of their involvement, the benefits and risks involved, and freedom of choice of participation in the study.

The inclusion criteria included age between 18 and 65 years, non-smoking status, and belonging to one of the following four categories: 1) patients with type 2 diabetes mellitus but without chronic kidney disease (T2DM), 2) patients with type 2 diabetes mellitus and proteinuric DKD (PDKD), 3) patients with type 2 diabetes mellitus and non-proteinuric DKD (NPDKD), and 4) healthy prospective living kidney donors (HC).

Exclusion criteria were the presence of any micro- or macrovascular complication in patients with the diagnosis of diabetes mellitus but without CKD, history or diagnosis of nephrolithiasis or urinary tract stones or any other kidney disease, present or past urinary tract infections, present or past diagnosis of malignancy, or life expectancy < 12 months or unstable clinical course over last 3 months as judged by treating physician.

Each participant's demographic and clinical details were recorded. Blood and urine biochemistry measurements were done on the AU5800 Clinical Chemistry Analyzer (Beckman Coulter, USA). Serum creatinine was measured by Modified Jaffe’s assay traceable to IDMS standards (total precision < 6% coefficient of variation). The urine protein was determined by pyrogallol red dye-based colorimetric assay (total precision < 5% coefficient of variation).

### Exosome isolation

Exosome isolation was divided into two phases- in vitro (from human kidney PTC, HK-2 cells) and in vivo (from human urine). The most common way to isolate exosomes from samples is to centrifuge them at high speed for at least 70 min at 100,000 to 200,000 × g^13^. Therefore, based on the studies performed by most of the researchers the following low and high-speed centrifugation was carried out for isolating exosomes.

HK-2 cells were procured from the American Type Culture Collection (ATCC, CRL 2190). HK-2 cells were cultivated in Keratinocyte Serum-Free Media (Gibco, USA) supplemented with epidermal growth factor (5 ng/ml) (Gibco, USA) and bovine pituitary extract (0.05 mg/ml) (Gibco, USA) as growth factors followed by 1% penicillin–streptomycin (MP Biomedicals, USA). They were treated with low glucose (LG; 5 mM) and high glucose (HG; 30 mM) for 96 h. Exosomes were isolated from these cells as described by Valadi et al*.*^14^ with minor modification. Briefly, the conditioned media (90 ml) was passed through two series of centrifugation-500 × g for 10 min followed by 16,500 × g for 30 min at 4 °C. The supernatant was centrifuged at high speed at 1,20,000 × g for 70 min at 4 °C. The pellet dissolved in PBS was filtered through a 0.1 µm syringe filter (EMD Millipore, USA) and washed with PBS at 1,20, 000 × g for 70 min at 4 °C. The pellet was resuspended in PBS with a protease inhibitor cocktail (PI) (1.67 ml of 100 mM sodium azide, 2.5 ml of 10 mM Phenylmethylsulphonyl fluoride (PMSF), 50 µl of 1 mM leupeptin for 50 ml of fluid) and stored at − 80 °C until use.

For the in vivo study, the second-morning mid-stream urine sample (100 ml) was collected from all the subjects in a sterile urine container with a PI cocktail and stored at − 80 °C until further use. To reduce variability between subjects based on diurnal patterns, second-morning urine collected from large bladder residues was used instead of first-morning urine^15^. Exosomes were isolated from urine as described by Patricia et al*.*^16^. Briefly, on the removal of urine samples from − 80 °C, they were vortexed extensively followed by centrifugation at 17,000 × g for 15 min at 4 °C. The supernatant was collected, and the pellet was resuspended in an isolation solution (250 mM sucrose, 10 mM Tri-ethanolamine, pH-7.6) following treatment with dithiothreitol (200 mg/ml, Sigma-Aldrich, USA). The resuspended pellet was again centrifuged in the same condition as mentioned above. All the supernatants were pooled and ultracentrifuged at 2,00,000 × g for 70 min at 4 °C. The pellet dissolved in PBS was filtered via a 0.1 µm syringe filter and washed with PBS at 2,00,000 × g for 70 min at 4 °C. The pellet was resuspended in PBS with PI and stored at − 80 °C until use.

### Flow cytometry analysis

Exosomes were incubated overnight at 4 °C with aldehyde sulfate latex beads (Invitrogen, USA) coated with purified anti-human CD63 (2 × 10^5^ CD63 coated beads/sample) (# 312002; BioLegend, USA). This complex was probed with PE anti-human CD81 (# 349505; BioLegend, USA)^17^, and data were acquired using FACSAria II (BD, Biosciences) followed by the data analysis in the FlowJo v10.8.1 trial version (Ashland, OR). A more detailed description of the coating of CD63 to the beads is provided in Supplementary Methods.

### Transmission electron microscopy (TEM)

Exosomes purified by differential centrifugation were loaded on Nickle carbon grids (TAAB Laboratories Equipment Ltd, England), negatively stained with 1% phosphotungstic acid, and viewed on Hitachi H7500-120 kV Electron Microscope^18^.

### Western blot

The exosomal and cellular samples were resuspended in RIPA Buffer (Sigma-Aldrich, USA) with PI and vortexed every 2 min for 30 min (on ice) followed by high spin at 4 °C at 14,000 × rpm for 15 min. The supernatant containing the protein was quantified by the Bicinchoninic Acid (Sigma-Aldrich, USA) assay method. 30 µg of protein (from HK-2 cells derived exosomes) and 2–3 mg of urinary protein (from urinary exosomes; normalized by urine creatinine) were resolved on 10% polyacrylamide gel electrophoresis, electrotransferred to polyvinylidene fluoride membrane (EMD Millipore, USA). The membrane was blocked with 5% skimmed milk for 2 h and probed sequentially for Tumor Susceptibility Gene (TSG) 101 (sc-7964, Santa Cruz Biotechnology, USA) and Calnexin (sc-11397, Santa Cruz Biotechnology, USA) overnight at 4 °C. Subsequently, the membranes were incubated for 90 min with the respective secondary antibodies. The membrane was covered by ECL solution (Clarity Max Western ECL Substrate, BioRad, USA) and visualized for the antibody binding in ChemiDoc XRS + (BioRad, USA). HK-2 cells were taken as a positive control.

### miRNA profiling

Total RNA was isolated from HK-2 cells derived exosomes by mirVana miRNA Isolation Kit (Invitrogen, United States) as recommended by the manufacturer. These samples were submitted to Exiqon (Vedbaek, Denmark) for miRNA real-time-based expression profiling. 19 μl RNA was reverse transcribed in 95 μl reactions using the miRCURY LNA Universal RT microRNA PCR, Polyadenylation, and cDNA synthesis kit (Exiqon). cDNA was diluted 50 × and assayed in 10 µl PCR reactions according to the protocol for miRCURY LNA Universal RT microRNA PCR; each microRNA was assayed once by qPCR on the microRNA Ready-to-Use PCR, Human panel I + II using ExiLENT SYBR Green master mix. Negative controls excluding template from the reverse transcription reaction were performed and profiled like the samples. The amplification was performed in a LightCycler 480 Real-Time PCR System (Roche) in 384 well plates. The amplification curves were analyzed using the Roche LC software, both for the determination of Ct (Cycle threshold) (by the 2nd derivative method) and for melting curve analysis. Using NormFinder, the best normalizer was found to be the average of assays detected in all samples. All data were normalized to the average of assays detected in all samples (average –assay Ct).

### miRNA validation

Total RNA was isolated from exosomes by mirVana miRNA Isolation Kit (Invitrogen, USA) with slight modification. 1 µl synthetic spike-in mix (UniSp2, UniSp4, UniSp5; RNA Spike-In Kit, Exiqon) per RNA preparation was added to 60 µl of lysis buffer to reduce the technical variance between the samples. This lysis buffer was added to the sample and vortexed vigorously for 10 min followed by the instructions mentioned in the mirVana miRNA Isolation Kit. RNA was eluted in 20 µl of RNase- free water.

Furthermore, determining RNA yield by spectrophotometric reading is frequently not achievable; instead, we employed RNA quantities based on starting volume in the PCR reaction as a measure, followed by spike-in quantification^19^. Cellular RNA was quantified with the Infinite 200 Pro NanoQuant microplate reader (Tecan, Männedorf, Switzerland). The reverse transcription reaction employed 4 µl of each RNA solution or 5 ng/µl of cellular RNA by using the Universal cDNA Synthesis Kit II (Exiqon) and UniSp6 RNA Spike-in template (to control for the potential inhibitors) according to the manufacturer's instructions. cDNA samples (diluted to 1:40) were probed by Exiqon primers for hsa-miR-155-5p (E24308), hsa-miR-28-3p (E24119), hsa-miR-425-5p (E24337), and hsa-miR-663a (E24284) via ExiLENT SYBR Green master mix (Exiqon) in a 7500 Real-Time PCR machine (Applied Biosystems, United States) with 40 amplification cycles. The cycling parameters were followed as recommended by Exiqon. Raw data were processed in 7500 software v2.0.1 to assign the baseline and threshold. We utilized spike-in UniSp2 and UniSp4 for normalization because there is no consistent endogenous control for exosomal miRNA^20,21^ while cellular RNA was normalized to U6 (E203950). The expression levels of differentially expressed miRNA were calculated as relative fold change^22^.

### miRNA target identification

The genes of the targeted miRNAs were scrutinized by the bioinformatic tool mirDIP v4.1 (http://ophid.utoronto.ca/mirDIP/) (confidence class as medium and prediction by at least three different algorithms) which integrates across 30 resources for miRNA-mRNA target prediction. These genes from mirDIP v4.1 were verified for their presence in the other four updated prediction tools—miRTarBase 7.0 (http://miRTarBase.mbc.nctu.edu.tw/), TarBase v.8 (http://www.microrna.gr/tarbase), miRWalk 3.0 (http://mirwalk.umm.uniheidelberg.de), and TargetScan 7.2 (http://www.targetscan.org/vert_72/). Genes predicted by at least three of the databases were considered speculative targets for the miRNA.

### IPA analysis

The plausible target genes were run for Core Analysis in IPA software (Ingenuity Systems, www.qiagen.com/ingenuity) to identify enriched ‘canonical pathways’ as well as ‘disease and functions’. Using right-tailed *Fisher's exact test*, a *p*-value was calculated for each biological function or disease assigned to that data set based strictly on chance. General settings, networks, and data sources were the same as the default software configuration. Supplementary File 1, Sheet 1 summarizes changes made in other filters used for this analysis. The genes from these pathways were connected using the information contained in Ingenuity Knowledge Database findings by the ‘Connect tool’. This filter was not changed except for the disease category that was selected as ‘endocrine system illnesses’ and ‘renal and urological diseases’. ‘Path Explorer’ tool was used to connect miR-663 and the genes of the network formed above. This tool had the same filters as the default set up by the program. The Molecule Activity Predictor (MAP) tool was used to find the association between miR-663 and genes in the network. This complete network was then overlaid with the ‘Disease and Function’ tool to determine the significant diseases and functions relevant to our study. The flowchart of the different tools utilized in IPA is illustrated in Supplementary Fig. S1.

### Statistical analysis

SPSS v22.0 for Windows (IBM SPSS, USA) was used for descriptive analysis of the variables from each patient group. RT-PCR graphs for both in vitro and in vivo were assessed by GraphPad Prism version 7.0 for Windows (GraphPad Software, San Diego, CA, USA, www.graphpad.com/). Differential expressions of miRNAs were analyzed by a two-tailed *Student t-test* or *Mann–Whitney* as appropriate. Relative expression of the PCR data was expressed in terms of fold change (2^- (delta delta Ct)).

In each group, we enrolled at least five patients with adequate urine samples using convenience sampling.

## Results

### Characterization of exosomes

**Figure 1:**
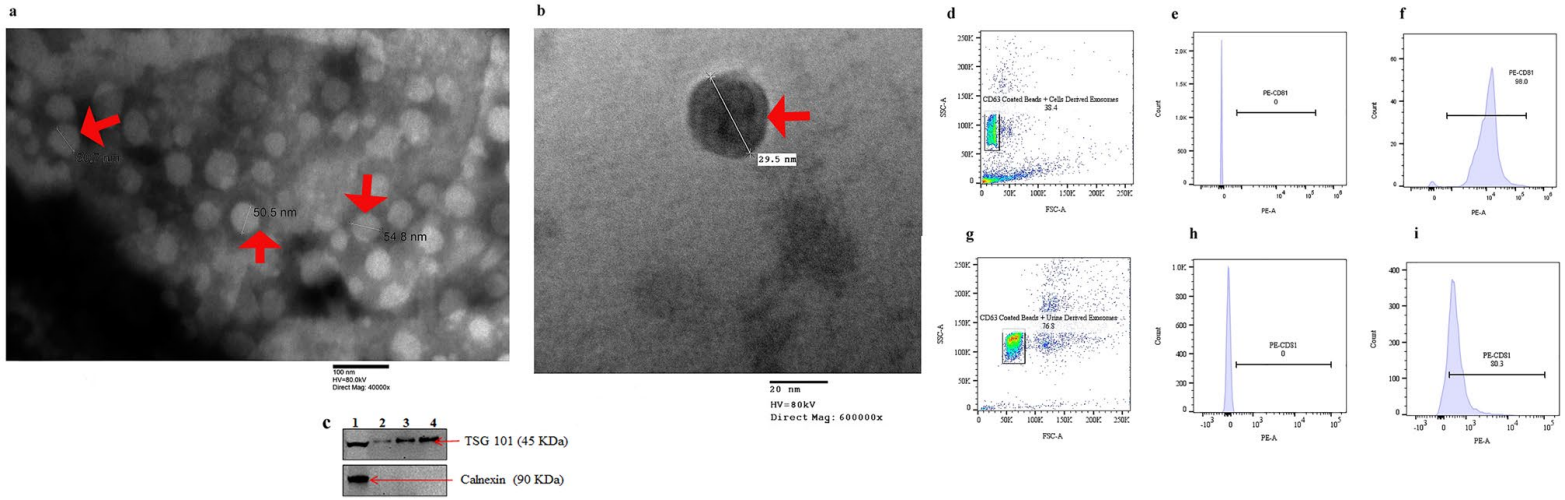
Characterization of exosomes. Electron microscopy of (**a**) HK-2 cells-derived exosomes. (**b**) Human urinary exosomes. The exosomes are shown by the red arrows. (**c**) Western blot of classical exosomal marker TSG 101 (exposure time: 120 s) and non-exosomal marker Calnexin (exposure time: 30 s); 1: HK-2 cells; 2,3: 2 and 3 mg of protein of healthy volunteer normalized to urine creatinine; 4: 30 μg of protein from HK-2 cells-derived exosomes. The original blots are presented in Supplemental Fig. S2. Scatter plot analysis of CD63 coated beads with (**d**) HK-2 cells-derived exosomes and (**g**) Human urine-derived exosomes. Gating of antibody bead conjugated exosomes derived from (**e**) HK-2 cells (**h**) human urine. The antibody bead exosome complex stained with anti-CD81 PE exhibited (**f**) 98% of positive CD81 vesicles derived from HK-2 cells. (**i**) Exosomes derived from human urine showed 80.3% of positive exosomes. TSG, Tumour Susceptibility Gene; SSC, Side Scatter; FSC, Forward Scatter; PE, Phycoerythrin.

TEM examination of HK-2 cells and human urine-derived exosomes revealed spherical vesicles of sizes 25–60 nm (Fig. 1a and b; red arrows) thereby confirming the presence of exosomes in the samples isolated. The protein bands of TSG 101 were expressed in HK-2 cells, human urine and HK-2 cells derived exosomes while calnexin, an endoplasmic reticulum marker was absent in all the samples except in HK-2 cells (Fig. 1c, Supplementary Fig. S2) Thus, this showed that the pellet collected after ultracentrifugation was indeed exosomes. The side/forward scatter showed the population of CD63-coated beads with cells-derived exosomes (Fig. 1d) and human urine-derived exosomes (Fig. 1g). The presence of known exosome marker CD81^23^ in the samples isolated as assessed by flow cytometry confirmed the successful isolation of exosomes from HK-2 cells (Fig. 1f) as well as human urine (Fig. 1i) in comparison to unstained controls (Fig. 1e and h) respectively.

### miRNA expression profiling of HK-2 derived exosomes under diabetic conditions

**Figure 2:**
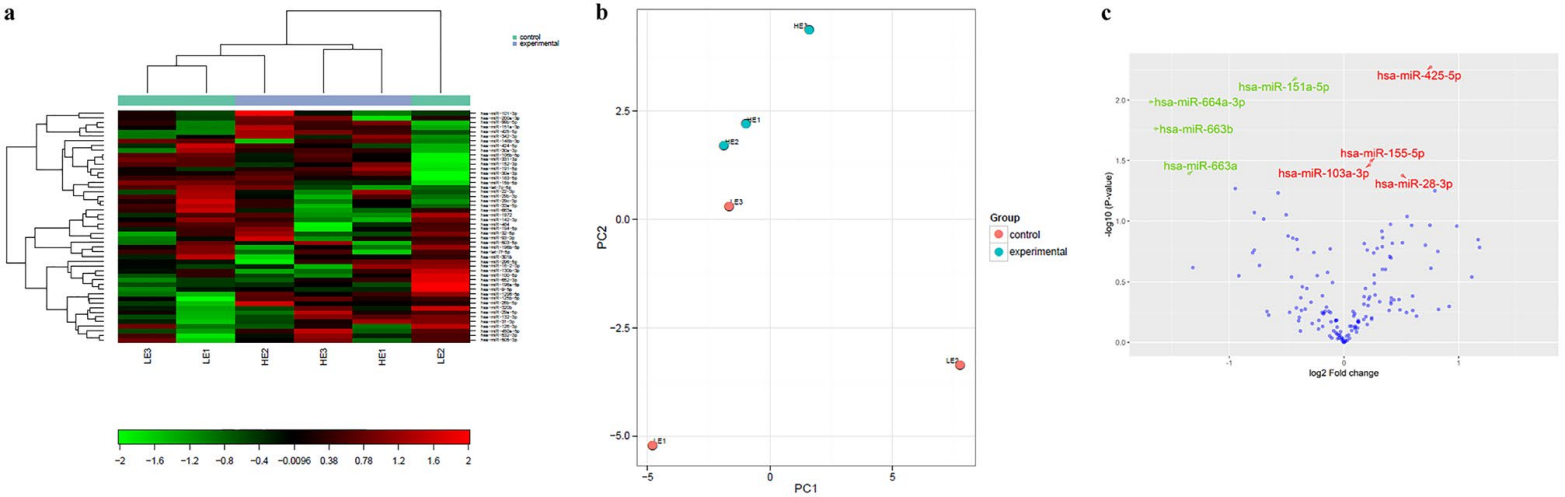
miRNA profiling by Real-Time PCR identified differentially expressed miRNAs in HK-2 derived exosomes under hyperglycaemia. (**a**) Heat map and unsupervised hierarchical clustering. Clustering was performed in all the samples for the top 50 miRNAs with the highest standard deviation. The color scale shown at the bottom illustrates the relative expression level of a microRNA across all samples: red color represents an expression level above the mean, and green color represents expression lower than the mean. (**b**) Principal Component Analysis was performed on all the samples and the top 50 miRNAs with the highest standard deviation. The normalized (dCt) values have been used for the analysis. (**c**) The volcano plot shows the relation between the *P*- values and the fold change. Highlighted spots are microRNAs with *p* < 0.05 after Benjamini–Hochberg correction for multiple testing. The significantly upregulated miRNAs are shown in red while downregulated miRNAs are shown in green color. miRNAs, Micro Ribonucleic Acid; PCA, Principal Component Analysis; LE1, LE2, LE3, Low glucose treated HK-2 cells-derived exosomes from three different samples (control); HE1, HE2, HE3, High glucose treated HK-2 derived exosomes from three different samples (experimental).

To mimic diabetes under in vitro system, the HK-2 cells were exposed to HG in comparison to the control i.e., LG. cDNA synthesis control UniSp6 was constant in LG (LE1, LE2, LE3) and HG (HE1, HE2, HE3) treated exosomal samples, showing no evidence of PCR inhibitors. (Supplementary Fig. S3A). A quality control assessment using miR-23a, miR-30c, miR-103, miR-142-3p, and miR-451 showed the quality and purity of the samples (Supplementary Table ST1, Supplementary Fig. S3B). For the differential miRNA profiling, the heat map (Fig. 2a) demonstrated a two-way hierarchical cluster analysis on LG (LE1, LE2, LE3) and HG (HG1, HG2, HG3) treated exosomal groups. Differential clustering by principal component analysis confirmed the distinction of the group (Fig. 2b). In the volcano plot, log2 (fold change) on X-axis and negative log *p*-value on Y-axis represented the differences in miRNA expression between HG and LG exosomes (Fig. 2c). Based on *Wilcoxon* or *t-test* as appropriate, eight miRNAs (*p* < 0.05) viz. hsa-miR-425-5p (1.7 fold), hsa-miR-155-5p (1.2 fold), hsa-miR-103a-3p (1.2 fold), hsa-miR-28-3p (1.4 fold) were upregulated while four miRNAs, hsa-miR-151a-5p (− 1.3 fold), hsa-miR-664a-3p (− 3.2 fold), hsa-miR-663b (− 3.1 fold), hsa-miR-663a (− 2.5 fold) were downregulated in HG as compared to LG treated HK-2 cells derived exosomes (Table 1).

**Table 1:** Differentially expressed miRNAs in HK-2 cells-derived exosomes under high glucose versus low glucose conditions.

miRNA name	Average dCt exp	Average dCt control	Fold change	*p-*value
hsa-miR-425-5p	− 0.89	− 1.6	1.7	0.0053
hsa-miR-151a-5p	0.39	0.81	− 1.3	0.0066
hsa-miR-664a-3p	− 5.8	− 4.1	− 3.2	0.010
hsa-miR-663b	− 0.42	1.2	− 3.1	0.017
hsa-miR-155-5p	2.7	2.4	1.2	0.032
hsa-miR-103a-3p	3.0	2.8	1.2	0.034
hsa-miR-663a	1.5	2.8	− 2.5	0.040
hsa-miR-28-3p	− 0.15	− 0.66	1.4	0.042

*p*-value was determined by *two tailed Student’s t-test* or *Wilcoxon test* as appropriate. miRNA, Micro Ribonucleic Acid; HK-2, Human Kidney Proximal Tubular Cells; hsa, Human; dCt, Normalized Threshold Values; exp, Experimental Sample (High glucose treated exosome sample); control, Low glucose treated exosome sample.

### Real-time PCR

**Figure 3:**
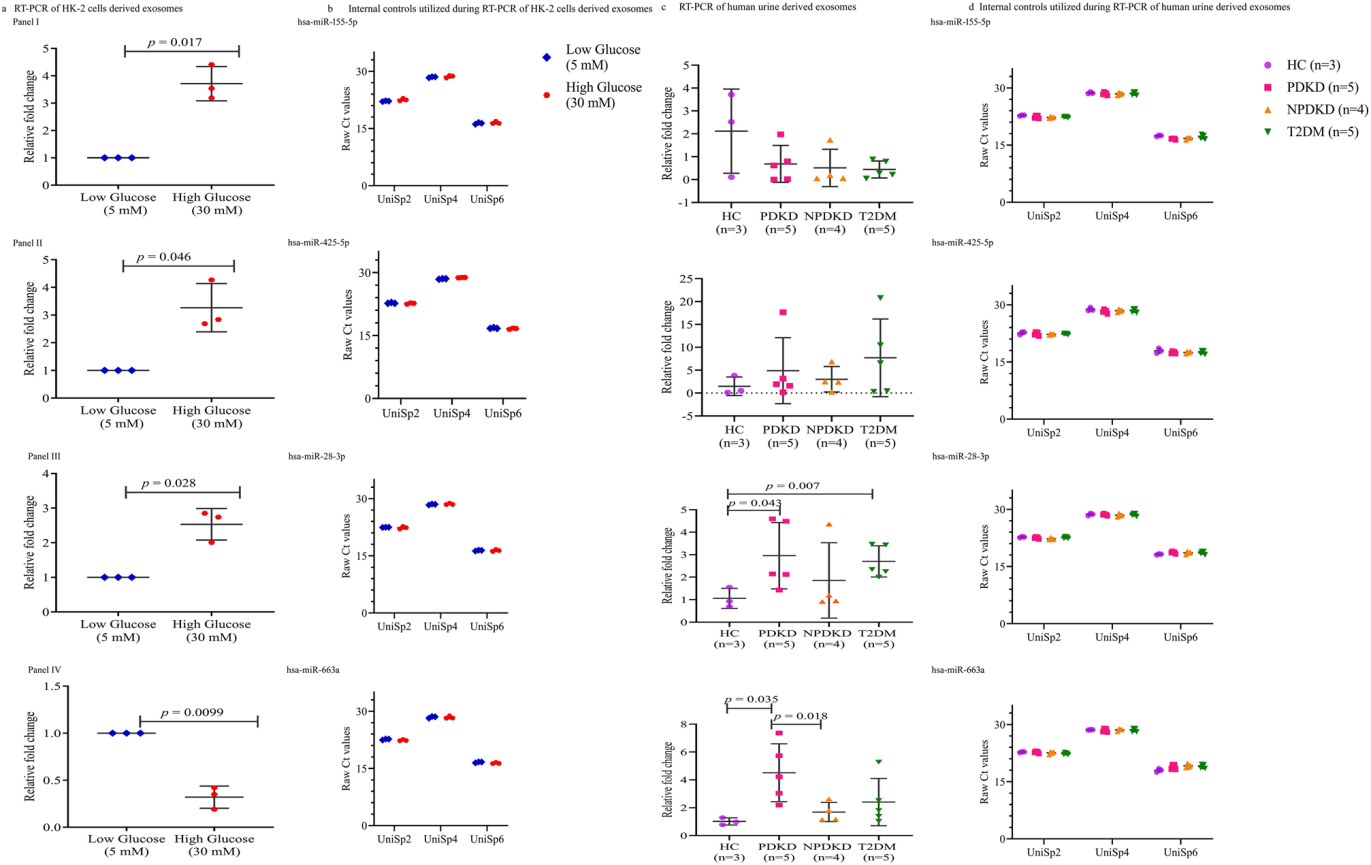
Validation of enriched miRNAs from HK-2 cells and human urine-derived exosomes by Real-Time PCR and their internal controls. hsa-miR-155-5p (Panel I), hsa-miR-425-5p (Panel II), hsa-miR-28-3p (Panel III) and hsa-miR-663a (Panel IV) were analyzed for their expression in (**a**) HK-2 cells-derived exosomes (n = 3). These cells were treated with 5 mM and 30 mM glucose for 96 h before exosome isolation. (**c**) Human urine-derived exosomes from HC, PDKD, NPDKD and T2DM. Relative fold change = (2^- (delta delta Ct)). UniSp2 and UniSp4 were used to estimate the RNA isolation quality; UniSp6 was used as a cDNA synthesis control for (**b**) HK-2 cells derived exosomes (**d**) human urine derived exosomes. In all scatter plots, the centre line represents the mean; the smallest number in the data set was shown at the end of the lower whisker; the largest number in the data set was shown at the end of the upper whisker as determined by the GraphPad Prism version.7.0 software. Normally distributed samples were analyzed by two-tailed *Student’s tests* otherwise by *Mann -Whitney* Test. Data is represented as Mean ± SD. hsa, *Homo sapiens*; Ct, Threshold; HC, Healthy Control; PDKD, Proteinuric Diabetic Kidney Disease; NPDKD, Non-Proteinuric Diabetic Kidney Disease; T2DM, Type 2 Diabetes Mellitus.

For microarray data validation, RT-PCR analysis of four miRNAs namely, hsa-miR-155-5p, hsa-miR-425-5p, hsa-miR-28-3p, and hsa-miR-663a from both in vitro and in vivo diabetic models were carried out. Significant overexpression was observed for hsa-miR-155-5p (*p* = 0.017, Fig. 3a, Panel I), hsa-miR-425-5p (*p* = 0.046, Fig. 3a, Panel II), hsa-miR-28-3p (*p* = 0.028, Fig. 3a, Panel III), while a significant downregulation was observed for hsa-miR-663a (*P* = 0.0099, Fig. 3a, Panel IV) in fresh batch of HG than to LG treated HK-2 cells-derived exosomes. The threshold values of the RNA spike-in controls (UniSp2 and UniSp4) and cDNA synthesis control (UniSp6) utilized during this experimental set up is represented in Fig. 3b. The miRNA profiling data also validated these observations (Table 1).

### Study population

For 6 months between January and June 2018, 60 patients were screened (Supplementary Fig. S4). 14 patients were excluded, and 11 patients refused to participate. Out of 35 participants who were enrolled in giving urine specimens, 10 participants either did not turn up for the scheduled visit or withdrew consent. Therefore, we had clinical details and adequate urine specimen collection for 25 participants (T2DM, n = 7; PDKD, n = 8; NPDKD, n = 5; HC, n = 5). By using spike-in controls to check for technical variability during RNA isolation, eight samples were excluded. Finally, the in vivo study consisted of 17 samples (T2DM, n = 5; PDKD, n = 5; NPDKD, n = 4; HC, n = 3) which were assessed for the targeted miRNA expression. The descriptive and clinical characteristics of the study groups are depicted in Table 2.

**Table 2:** Demographic and clinical parameters of the study groups.

Parameters	Proteinuric Diabetic Kidney Disease (PDKD; n = 5)	Non-Proteinuric Diabetic Kidney Disease (NPDKD; n = 4)	Type 2 Diabetes Mellitus (T2DM; n = 5)	Healthy Volunteers (Control; n = 3)
Age (years)	55.40 ± 8.01	58.0 ± 4.08	54.2 ± 8.78	44.33 ± 5.85
Sex (Male/Female) (%)	60/40	75/25	20/80	33.3/66.7
Duration of diabetes (months)	156.40 ± 87.57	111.00 ± 73.56	108.00 ± 20.78	NA
Duration of kidney problem (months)	22.80 ± 16.10	16.25 ± 13.28	NA	NA
Fasting blood glucose (mg/dl)	131.80 ± 49.44	137.65 ± 38.64	134.50 ± 39.10	89.66 ± 1.52
Serum creatinine (mg/dl)	1.69 ± 0.63	1.89 ± 0.53	0.81 ± 0.14	0.5 ± 0.06
eGFR	44.84 ± 14.92	39.17 ± 15.36	86.06 ± 14.39	122.07 ± 4.23
24 h urine protein (mg/dl)	3059.44 ± 2003.71	86.80 ± 41.72	94.35 ± 32.33	110.60 ± 34.73

Data are shown as Mean ± Standard Deviation. NA, not applicable; mg/dl, milligrams/deciliter.

The same four miRNAs, as validated in HK-2 derived exosomes, were also verified for their expression levels in these groups (Fig. 3c). The hsa-miR-663a was significantly downregulated in NPDKD than to PDKD (*p* = 0.018) and elevated in PDKD than to the HC (*p* = 0.035). The hsa-miR-663a was found to be expressed at a higher level in T2DM as compared to NPDKD, though data was statistically non-significant (Fig. 3c, Panel IV). The hsa-miR-28-3p was significantly elevated in PDKD (*p* = 0.043) and T2DM (*p* = 0.007) than to HC (Fig. 3c, Panel III). The hsa-miR-425-5p (Fig. 3c, Panel II) was upregulated while has-miR-155-5p was decreased (Fig. 3c, Panel I) in all the groups i.e., PDKD, NPDKD, and T2DM than to the HC (non-significant) The expression level of different miRNAs varied with variable levels of albuminuria in the affected individuals. The internal controls (UniSp2, UniSp4 and UniSp6) threshold values are represented in Fig. 3d.

### Target genes identification for miR-663

**Figure 4:**
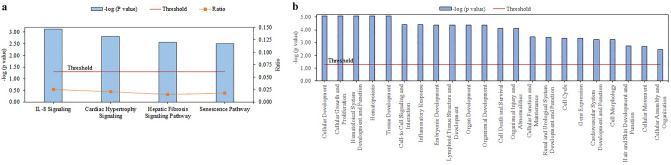
Core Analysis of miR-663a target genes by Ingenuity Pathway Analysis Software. The plausible targeted genes of miR-663a were analyzed for Core Analysis by Ingenuity Pathway Analysis software which yielded (**a**) 4 enriched canonical pathways. (**b**) Significant 22 diseases and functions. Blue bars represent the *p-*value for each pathway as well as disease and functions and are expressed as − log (*p-*value). The threshold line corresponds to 0.05 (red line). The ratio (orange points) on each pathway represents the ratio of the number of genes from the dataset that meet the cut-off criteria divided by the total number of genes that map to that pathway from the Ingenuity Knowledge Database. *p* < 0.01 and number of molecules ≥ 5 were the criteria for selecting canonical pathways as well as disease and functions.

We performed an *in-silico* analysis of the genes for miR-663a, as it was the only miRNA that showed differential expression between NPDKD and PDKD. These genes were analyzed by IPA for pathways and functional analysis as well as network construction. IPA retrieved 104 canonical pathways (Supplementary File 1, Sheet 2) and 51 diseases and bio functions (Supplementary File 1, Sheet 3) for these plausible genes (*p* < 0.05). Stringent conditions (*p* < 0.01; the number of molecules >  = 5) yielded four significant pathways namely IL-8 Signaling, Cardiac Hypertrophic Signaling, Hepatic Fibrosis Signaling, and Senescence Pathway (Fig. 4a) and 22 annotations under the diseases and bio functions tool (Fig. 4b).

The top five significant cellular and molecular function identified were cellular development, cellular growth and proliferation, cell to cell signaling and interaction, cell death and survival, and cellular function and maintenance. The ‘inflammatory response’ and ‘organismal injury and abnormalities’ were the most significantly enriched disease in the present study. To detect the association of kidney injury with ‘organismal injury and abnormalities’, the ‘diseases and functions’ annotation displayed apoptosis of podocytes and permeability of microvascular endothelial cells. Both these abnormalities were associated with DKD^24^. Similarly, ‘inflammatory response’ annotated antibody-dependent cell-mediated cytotoxicity (ADCC) of natural killer (NK) cells as its only function. IL-8 signaling, the top-ranked canonical pathway coincided well with the top-ranked disease i.e., inflammatory response.

**Figure 5:**
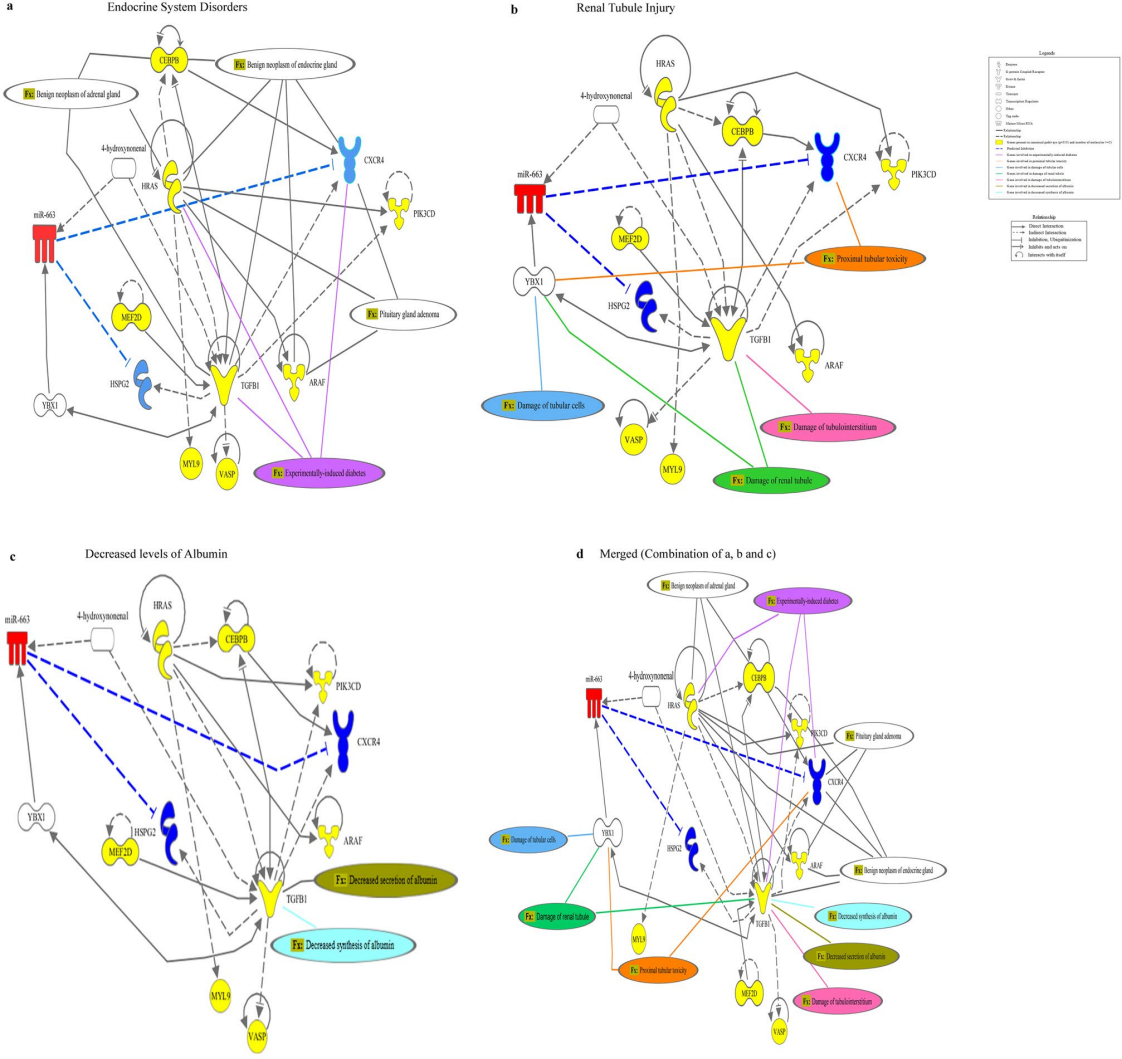
Disease and function analysis of the network by Ingenuity Pathway Analysis. The topological network created by Ingenuity Pathway Analysis Software when overlaid by the ‘Disease and Function’ tool generated (**a**) Endocrine System Disorders (*p* = 1.53E−07–3.89E−5). Genes involved in experimentally induced diabetes were—CXCR4, HRAS, and TGF-β1 (purple line). (**b**) Renal Tubule Injury (*p* = 5.76E−04–9.09E−03). This included sub-categories which were—Damage to tubule cells which consisted of gene YBX1 (sky blue line); Damage to renal tubules which involved genes YBX1 and TGF-β1 (green line); Damage of tubulointerstitium indicated TGF-β1 gene (pink line) and proximal tubular toxicity pointed CXCR4 and YBX1 gene (orange line). (**c**) Decreased levels of albumin (*p* = 2.28E−03–3.04E−3) included decreased synthesis (cyan line) and decreased secretion of albumin (light green line) with TGF-β1 as the central gene for both. ‘Molecule Activity Predictor’ predicted the relationship between miR-663 and CXCR4 (Reference no. 47) as well as miR-663 and HSPG2 (Reference no. 48) to be inhibited when the miR-663 expression was elevated based on the research findings of Ingenuity Knowledge Database. (**d**) Complete network showing Endocrine System Disorders, Renal Tubule Injury, and Decreased levels of albumin altogether. All molecules are represented in different shapes which are the default icon in Ingenuity Pathway Analysis software and all details of color-coded lines are mentioned in the legend. miR, Micro Ribonucleic Acid; HRAS, Harvey Rat Sarcoma Viral Oncogene Homolog; CEBPB, CCAAT/Enhancer-Binding Protein (C/EBP), beta; ARAF, A-RAF Proto-Oncogene, Serine/Threonine Kinase; PIK3CD, Phosphatidylinositol-4,5-bisphosphate 3-kinase, catalytic subunit delta; CXCR4, C-X-C Chemokine Receptor 4; TGF-β1, Transforming Growth Factor-Beta 1; MEF2D, Myocyte Enhancer Factor 2D; VASP, Vasodilator-Stimulated Phosphoprotein; MYL9, Myosin, Light Chain 9, regulatory; HSPG2, Heparan Sulfate Proteoglycan 2; YBX1, Y-Box Binding Protein 1.

We built a network of relationships between the genes of these enriched pathways and the other genes of the Ingenuity Knowledge Database (IKDB) which was further linked to miR-663 by the shortest route analysis (Supplementary File 1, Sheet 4). To build a network of associations, the top five routes were mapped (Supplementary Fig. S5, Supplementary File 1, Sheet 5). Interestingly, this network focused on the three major DKD-associated ‘diseases and functions’ relevant to our study—endocrine system disorders (Fig. 5a), renal tubule injury (Fig. 5b), and decreased levels of albumin (Fig. 5c). These data suggested that the genes in the proposed network would facilitate the pathogenesis of the DKD (Fig. 5d).

## Discussion

To the best of our knowledge, this is the first study wherein, differential miRNA expression profiling was identified in HK-2 cells derived exosomes under hyperglycemic conditions. Among the four shortlisted human miRNAs i.e., miR-155-5p, miR-425-5p, miR-28-3p, and miR-663a, only miR-663a expression was different between NPDKD and PDKD subjects. miR-155-5p, miR-425-5p, and miR-28-3p were also associated with T2DM and regulated insulin signaling^25,26^ which were validated in our experimental setup. However, variability in the expression of miR-155-5p, and miR-425-5p (non-significant) in the human urines of DKD excluded their further analysis. Though the source of isolation varied, increased expression of miR-425-5p was observed in the plasma and peripheral blood mononuclear cells of diabetic subjects than in controls^27^. While for miR-28-3p, which was up-regulated in our study and was increased in urine EVs of type 1 diabetics^28^. Since it has already been pointed out as a predictor of T2DM^29^, the focus was thus inclined towards miR-663a.

This indeed is the most intriguing finding of our study discovering miR-663a as an indirect reflection of mechanism between PDKD and NPDKD patients. A solitary study related to this miRNA reiterates miR-663a down-regulation in the kidneys of hypertensive patients as compared to normotensive males^30^.

Proteinuria is generally considered for diagnosing DKD, however, a proportion of patients develop renal impairment before albuminuria^3^. Under pathological conditions pattern of miRNA differs from the control and being secretory, they possess a strong potential with diagnostic value^31^.

Interestingly, miR-663a was significantly upregulated (*p* < 0.0001) in HK-2 cells while downregulated (*p* = 0.0099) in HK-2 cells derived exosomes (Supplementary Fig. S6). Further, the significant differential expression of miR-663a observed between the NPDKD and PDKD led to the investigation of pathways and networks regulated by this miRNA. The network analysis, through IPA, showed that miR-663a and its plausible target genes were involved in the pathogenesis of DKD with PTC being the primary contributors (Fig. 5d). Therefore, it provides the first line of evidence for miR-663a’s involvement in DKD. Bioinformatics analysis revealed 60 targets (Supplementary File 1, Sheet 6) by the intersection of five databases. Analyzing these genes by IPA uncovered the ‘canonical pathways’ and ‘disease and functions’ of miR-663 which were extrapolated in a topological network.

IL-8, a pro-inflammatory cytokine signaling turned out to be the most ‘enriched pathway’ and ADCC of NK cells was the only function of the top-ranked disease i.e., inflammatory response. Under HG, increased secretion of IL-8 with tubular injury was observed in human renal PTC^32^. Its urinary levels were also found to be elevated in the early stage of type 2 DKD^33^. NK Group 2D receptor known to protect the host against infections was aberrant in type 1 diabetics which were involved in its pathogenesis^34^, highlighted the connection of miR-663a target genes with the dysfunctional pathways and other complications related to DKD. For example, diabetes caused more pronounced fibrosis in the heart and liver^35^. Exposure of PTC to HG resulted in accelerated senescence. A similar expression was observed in the PTC at the early stages of type 2 DKD patients^36^. These studies validated our approach for the identification of genes and pathways connected with DKD paving the way for developing potential therapeutic interventions owing to PTC injury. The genes of these pathways were mapped to form an association among themselves by several direct and indirect connections and linked to miR-663. The observations pointed to endocrine system disorders, renal tubule injury, and decreased levels of albumin as the enriched ‘diseases and functions’.

Under endocrine disorder ‘experimentally induced diabetes’ was linked to Harvey Rat Sarcoma Viral Oncogene Homolog (H-RAS), C-X-C motif chemokine receptor 4 (CXCR4) and transforming growth factor-beta 1 (TGF-β1). All these genes were elevated in DKD^37,38^.

The renal tubular injury had four sub-divisions. The first category included ‘damage of renal tubule’ which was related to Y-box binding protein 1 (YBX1) and TGF-β1. The second category ‘damage to tubular cells’ was associated with YBX1. ‘Proximal tubular toxicity’, the third sub-division pointed towards YBX1 and CXCR4. TGF-β1 impaired renal tubular cell integrity leading to tubular atrophy, a hallmark of tubule-interstitial fibrosis, thereby damaging tubule-interstitium, represented the fourth category^39^. In various CKD mouse models and human PTC, CXCR4 was increased^40^ which in turn upregulated TGF-β1^41^ thereby promoting tubular injury and renal fibrosis in DKD^42^. In A459 cells, TGF-β1 increased the expression of YBX1 which regulated epithelial-mesenchymal transition (EMT)^43^, a common insult to PTC in diabetes^44^. Upregulation of rat Myb1a (YBX-1) and LCR1 (CXCR4) was also associated with the proximal toxicity in the male rat^45^.

For ‘decreased levels of albumin’, TGF-β1 was the central gene. HG elevated TGF-β1 which reduced the lysosomal activity, thereby reducing the processing of albumin by the kidney. Therefore, TGF-β1 regulated the uptake of albumin^46^. Overall, the network showed an inhibitory relationship between CXCR4 and heparan sulfate proteoglycan 2 (HSPG2) with miR-663 based on the findings from IKDB. Upregulated miR-663 inhibited CXCR4 in glioblastoma, thereby, attenuating the oncogenic properties^47^. This was complemented through an observation showing elevated miR-663 regulated chemoresistance by decreasing HSPG2 expression in breast cancer cells^48^.

**Figure 6:**
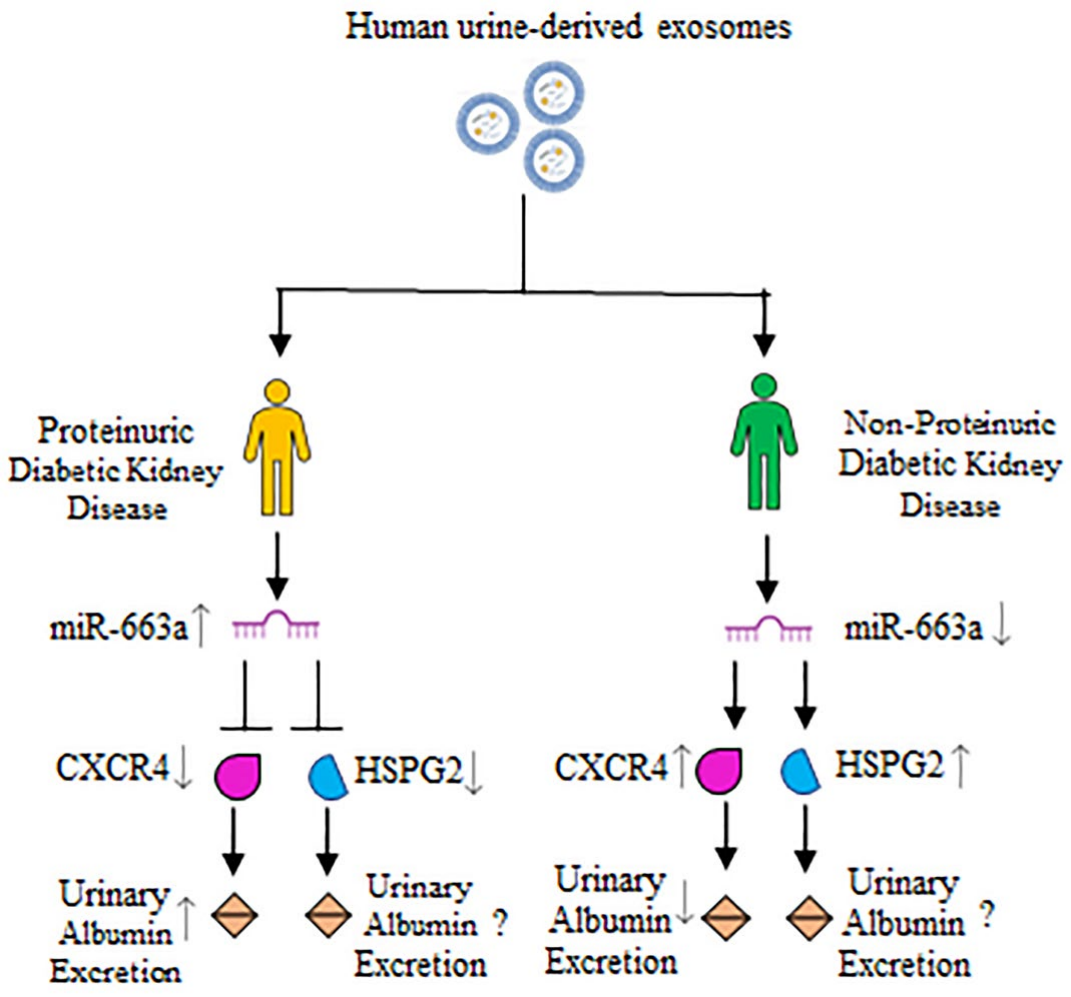
Schematic representation of the proposed hypothesis of the study. According to IPA, increased miR-663a expression reduced CXCR4 and HSPG2 expression (Fig. 6d). As a result, it is possible that elevated miR-663a expression in PDKD reduced CXCR4 expression, leading to albuminuria. Reduced HSPG2 expression was inversely associated with the severity of proteinuria. In the lack of heparan sulfate binding sites, however, there is no proteinuria, but the development of DKD is probable^51^. Reduced expression of miR-663a in NPDKD would increase CXCR4 and HSPG2 expression. As a result, albuminuria in these groups of subjects would be reduced. miR, Micro Ribonucleic Acid; CXCR4, C-X-C Motif Chemokine Receptor 4; HSPG2, Heparan Sulfate Proteoglycan 2.

Taking these cues, we hypothesize that decreased level of miR-663a might elevate the expression of CXCR4 and HSPG2 (Fig. 6) in non-proteinuric DKD. CXCR4 was upregulated in the biopsy of DKD patients and the experimental model which upon inhibition caused increased urinary albumin excretion and enhanced the proximal tubular cell death^49^. It would be noteworthy to verify this relationship between PDKD and NPDKD. Additionally, there might be a possibility that miR-663a increased expression observed in PDKD would reduce the CXCR4 expression resulting in albuminuria in PDKD, which warrants additional study. Decreased expression of heparan sulfate observed in the biopsy of type 2 overt DKD patients correlated inversely with the degree of proteinuria^50^. There could be a possibility of such phenomena occurring in PDKD. Contrarily, no proteinuria was detected in mice lacking heparan sulfate binding sites but the onset of the progression of DKD was observed^51^. Therefore, segregation of the patients into PDKD and NPDKD would aid in achieving better mechanistic approach for heterogenous Type 2 DKD.

Exosomes may have different miRNA profiles than their parents, which could explain the differential expression of miR-663a in cells and exosomes observed under HG. Therefore, cells may have an active exosome and cargo selection process^52^. One of the RNA binding proteins implicated in miRNA sorting in exosomes was YBX-1^53^. YBX-1 was shown to be related to miR-663a in this study as well, according to IPA analysis (Fig. 5d), implying that YBX1 might be involved in the differential expression of miR-663a observed in cells and exosomes in this experimental setup. The major limitation of this study is the small sample size. The study was exploratory in nature and no preliminary data was available in this regard. The results of this pilot exploratory study need to be validated in a larger cohort of patients with DKD.

We did not include patients with proteinuric or non-proteinuric CKD other than diabetes mellitus and hence, are not able to infer whether the same observations would be present outside a diagnosis of diabetes mellitus or not. Therefore, we cannot conclusively say that differential expression of miR-663a in proteinuric versus non-proteinuric CKD is exclusive to DKD.

Because bio-fluids contain less amount of total RNA, it is challenging to determine the quality and concentration of total RNA isolated for extracellular miRNA research. Furthermore, in a disease condition, a greater number of miRNAs can be released extracellularly than in a healthy state. To obtain accurate findings for biomarker detection studies, it is recommended to use an equivalent volume of starting material (serum, plasma, or any other biological fluid) rather than the same amount of total RNA^54,55^.

As mentioned by Nowak et al. PTC are dominant cells to be injured in non-proteinuric subjects and the fact that miR-663a expression was down-regulated significantly under HG both in vitro and in vivo in this study, might also suggest the involvement of PTC in the non-proteinuric subjects. But it cannot be denied that urine is composed of all the cells of the nephron, hence which cells from urine are predominant in NPDKD needs to be further investigated. Nevertheless, the results need to be explored further to identify the underlying mechanism between these two subsets of patients existing within DKD.

In closing, the expression of miR-663a in urine exosomes may be reflective of underlying mechanistic differences in kidney involvement in PDKD versus NPDKD. Analyses of the likely genes associated with miR-663a highlighted the significance of tubular cells in the pathophysiology of DKD. These observations need validation and further exploration in larger and diverse populations.

## Supplementary Information

Supplementary Figure S1.

Supplementary Figure S2.

Supplementary Figure S3.

Supplementary Figure S4.

Supplementary Figure S5.

Supplementary Figure S6.

Supplementary Information.

## Data Availability

The data discussed in this publication have been deposited in NCBI’s Gene Expression Omnibus and are accessible through GEO Series accession number GSE180411 (https://www.ncbi.nlm.nih.gov/geo/query/acc.cgi?acc=GSE180411). The lead author, Nisha Sinha (nishasinha04.pgi@gmail.com), can be contacted for access to the datasets created during and/or analyzed during the current study.
