# Supplementary figures and images for: Urinary exosomal miRNA-663a shows variable expression in diabetic kidney disease patients with or without proteinuria
Source: Sci Rep. 2023 Mar 18;13:4516. doi: 10.1038/s41598-022-26558-4 (PMC10024703; doi:10.1038/s41598-022-26558-4)

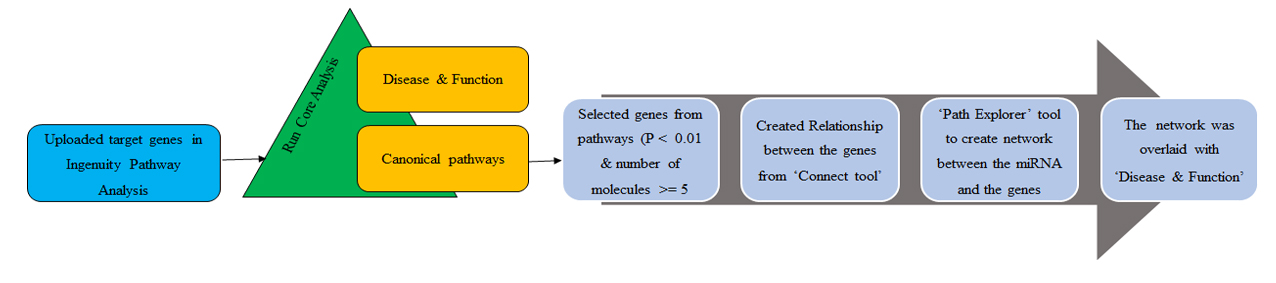

Supplement: Supplementary file 1 — Supplementary Figure S1. [file 41598_2022_26558_MOESM1_ESM.jpg]

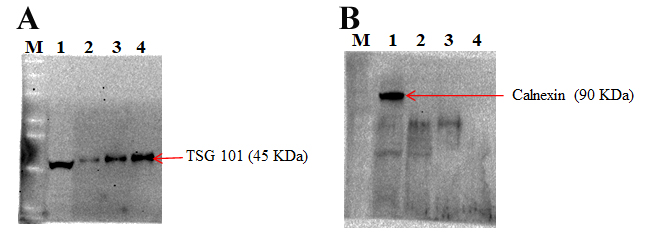

Supplement: Supplementary file 2 — Supplementary Figure S2. [file 41598_2022_26558_MOESM2_ESM.jpg]

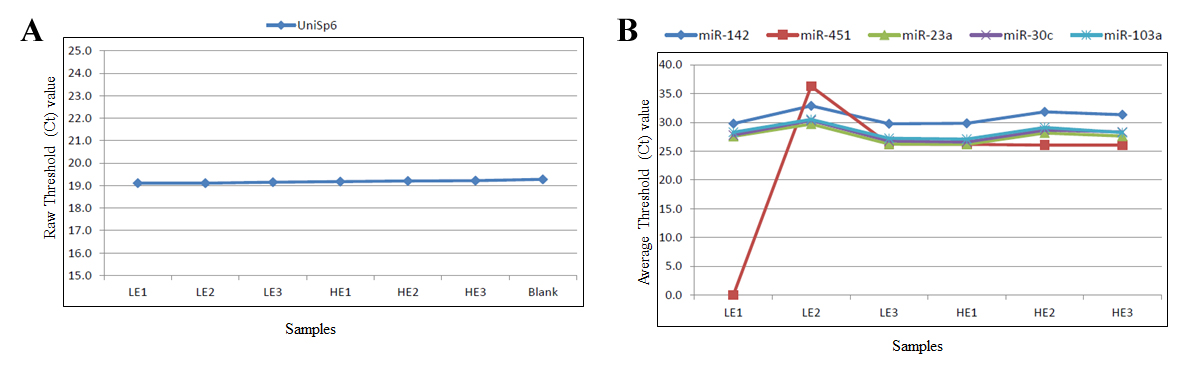

Supplement: Supplementary file 3 — Supplementary Figure S3. [file 41598_2022_26558_MOESM3_ESM.jpg]

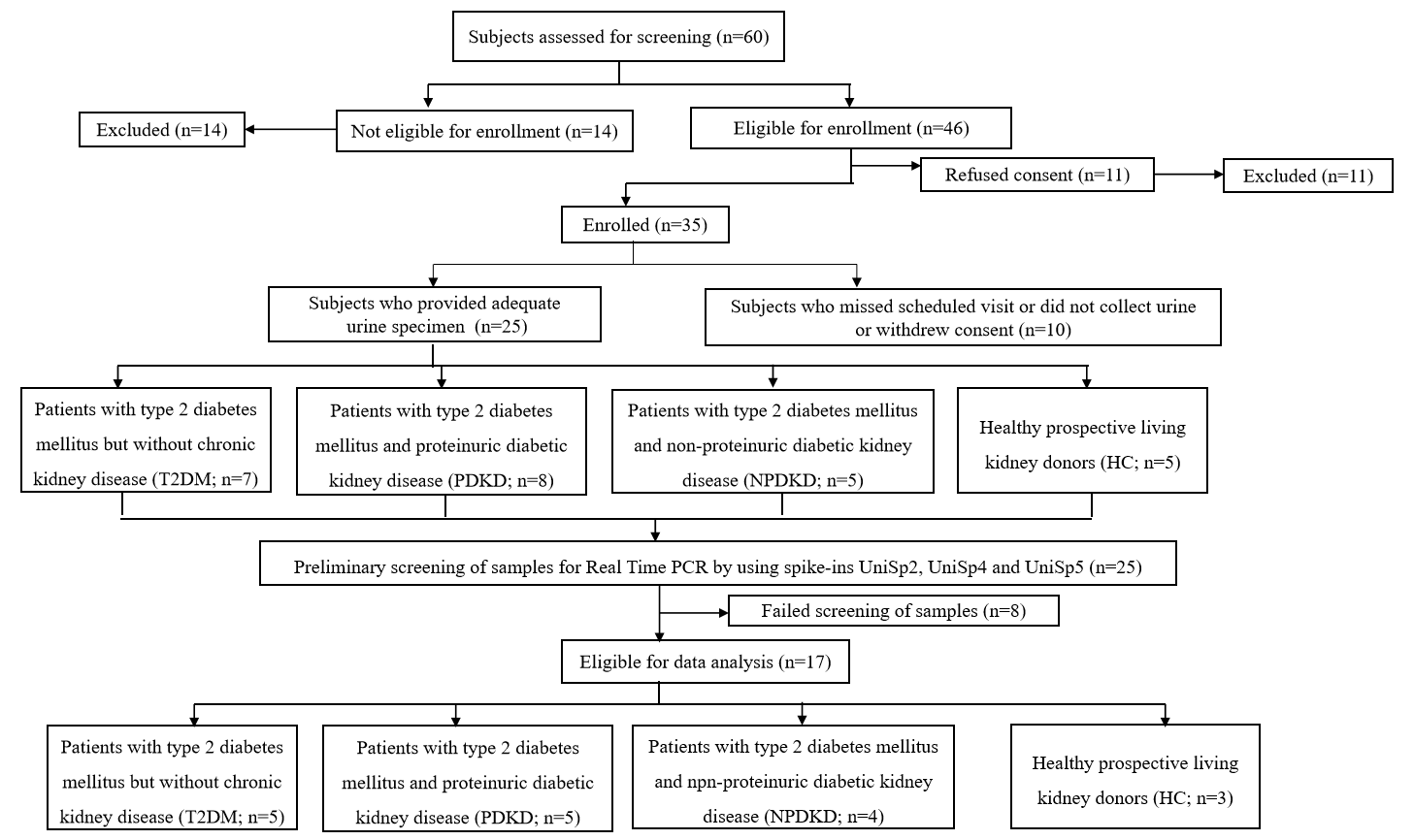

Supplement: Supplementary file 4 — Supplementary Figure S4. [file 41598_2022_26558_MOESM4_ESM.tif]

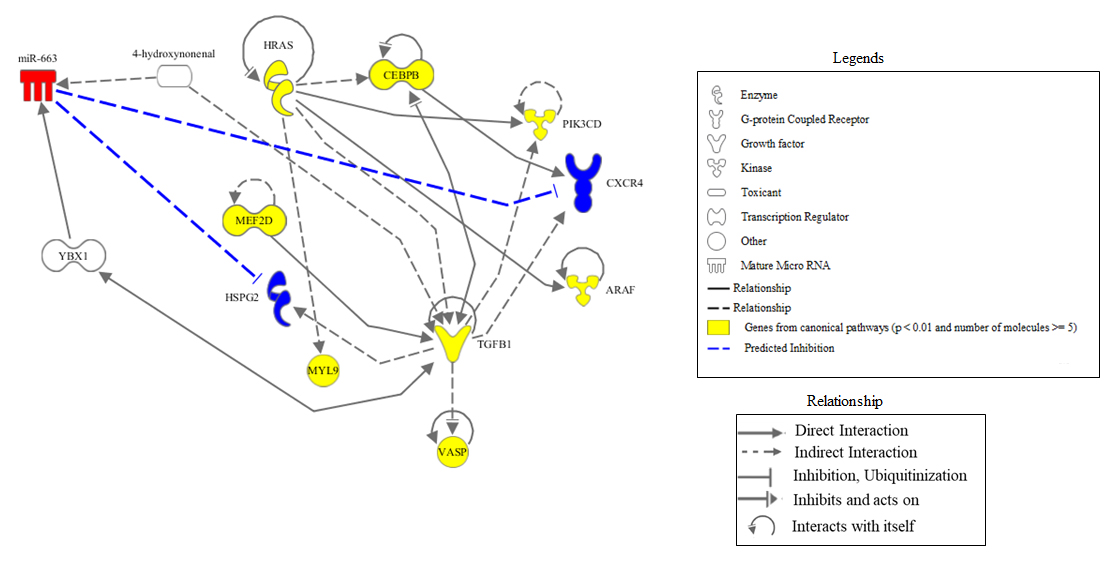

Supplement: Supplementary file 5 — Supplementary Figure S5. [file 41598_2022_26558_MOESM5_ESM.jpg]

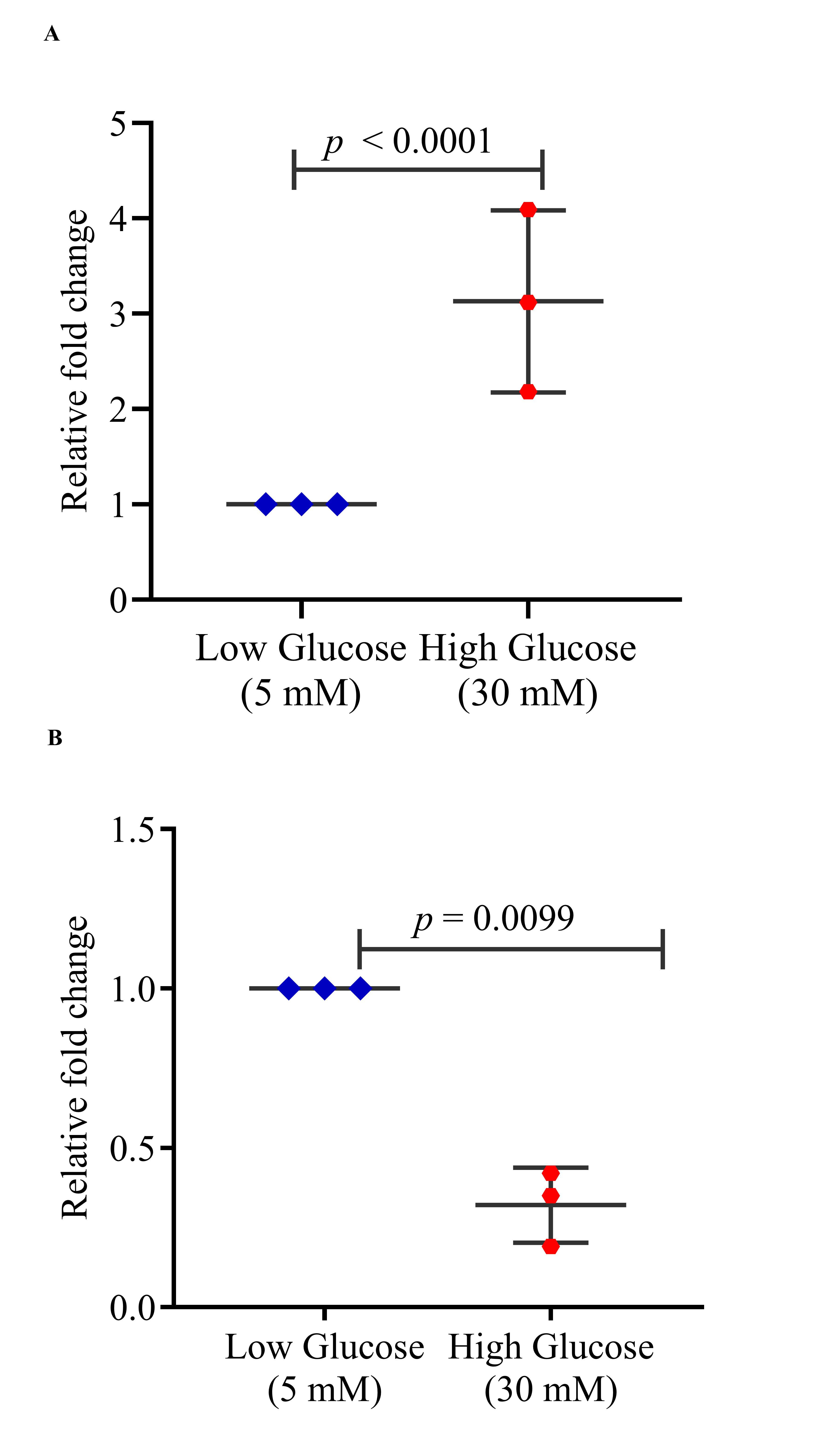

Supplement: Supplementary file 6 — Supplementary Figure S6. [file 41598_2022_26558_MOESM6_ESM.jpg]
