# Supplementary material for: Urinary exosomal miRNA-663a shows variable expression in diabetic kidney disease patients with or without proteinuria
Source: Sci Rep. 2023 Mar 18;13:4516. doi: 10.1038/s41598-022-26558-4 (PMC10024703; doi:10.1038/s41598-022-26558-4)
Supplement: Supplementary file 7 — Supplementary Information. [file 41598_2022_26558_MOESM7_ESM.pdf]

Urinary exosomal miRNA-663a differentiates proteinuric from non-proteinuric Diabetic Kidney Disease

Nisha Sinha[1, 3], Veena Puri[2], Vivek Kumar[3], Ritambhra Nada[4], Ashu Rastogi[5], Vivekanand Jha[6*], Sanjeev Puri[7*]

[1]Centre for Stem Cell Tissue Engineering and Biomedical Excellence, Panjab University, Chandigarh, India

[2]Centre for Systems Biology & Bioinformatics, Panjab University, Chandigarh, India

[3]Department of Nephrology, Post Graduate Institute of Medical Education & Research, Chandigarh, India

[4]Department of Histopathology, Post Graduate Institute of Medical Education & Research, Chandigarh, India

[5]Department of Endocrinology and Metabolism, Post Graduate Institute of Medical Education & Research, Chandigarh, India

[6*]The George Institute for Global Health, New Delhi, India

[7*]Department of Biotechnology, University Institute of Engineering & Technology (UIET), Panjab University, Chandigarh, India

Supplementary Methods

Antibody coating of the aldehyde sulfate beads

To 100 µl of (2-[N-Morpholino]ethanesulfonic acid) (MES) (Sigma-Aldrich, USA) buffer, 25 µl of 4 µm aldehyde sulfate latex beads (4 % w/v; Invitrogen, USA) was added. This mixture was centrifuged for 15 minutes (mins) at 3,000 x g. The pellet was dissolved in 100 µl of MES buffer and centrifuged for 20 mins at the same speed. 12.5 µg purified anti-human CD63 antibody (#312002; BioLegend, USA) was combined with 25 µl MES buffer. The beads were added to the antibody solution, which was then incubated at 4^0 C overnight with gentle agitation. The complex was centrifuged at 3,000 x g for 20 mins the next day. The pellet was dissolved in 100 µl of 1X PBS before being centrifuged for 20 mins at 3000 x g, which was done twice. The beads were dissolved in 100 µl of PBS storage buffer before being kept at 4^0 C for future use.

Coating of exosomes and flowcytometric analysis

Exosomes were combined with anti-CD63 beads (2 x 10^5 CD63 beads/sample) and incubated at room temperature for 15 mins. With 1X PBS, the capacity was increased to 500-1,000 µl. On a rotator, this entire combination was incubated overnight at 4^0 C. The reaction was stopped for 30 mins at room temperature by adding 100 mM glycine. This was washed twice in 500-1,000 µl of washing buffer (1X PBS + 1% BSA + 0.05 percent sodium azide) at 3,000 x g for 10 mins at room temperature. Exosome-coated beads were combined with phycoerythrin (PE) labelled CD81 antibody (#349505; Biolegend, USA) and gently shaken for 1 hour. The pellet was resuspended in 200 µl of 1X PBS and flow cytometry analysis was performed after the washing process was done twice.

Supplementary Figure Legends

Supplementary Figure 1. Flowchart for the progressive steps applied in Ingenuity Pathway Analysis software for creating the network associated with miR-663. The filters applied (if any) in any of the stages have been mentioned in the main article under IPA in Methods.

Supplementary Figure 2. Protein expression of HK-2 cells and human urine-derived exosomes. (A) Classical marker TSG 101 of exosomes was observed in human urine and HK-2 cells-derived exosomes. (B) Calnexin, an endoplasmic reticulum marker was absent in all the samples except the HK-2 cell. M: Puregene Prestained Protein Ladder; 1: HK-2 cells; 2-3: 2 and 3 mg of urinary exosomal protein normalized to urinary creatinine from healthy subjects; 4: 30 µg of exosomal protein from HK-2 cells derived exosomes

Supplementary Figure 3. Quality control of HK-2 cells derived exosomal sample. (A) cDNA synthesis control assays. The cDNA synthesis control (UniSp6) was added to the reverse transcription reaction to evaluate the reverse transcription reaction. The graphs show raw Ct obtained for the RNA spike-in assay. (B) The graph shows the average Ct obtained by all the samples for miR-142, miR-451, miR-23a, miR-30c and miR-103a. The X-axis denotes the samples used for miRNA profiling and Y-axis indicated the Ct value. Ct, Threshold Value; LE1, LE2, LE3 are three different low glucose treated HK-2 cells-derived exosomes; HE1, HE2, HE3 are three different high-glucose treated HK-2 derived exosomes.

Supplementary Figure 4. Flowchart depicting the screening and enrollment of diabetic kidney disease patients.

Supplementary Figure 5. Gene interaction network creation by Ingenuity Pathway Analysis (IPA) Software. Genes from the four significant pathways **(Figure 4)** were used to generate a topological network. These genes are shown in yellow color in the figure. 'Path Explorer' filter

from IPA was used to calculate the shortest distance between these genes and miRNA-663 which was overlaid with the 'Molecule Activity Predictor' filter to predict the upstream and downstream regulators. miRNA was upregulated (red color) which was predicted to inhibit CXCR4 and HSPG2 (blue color) based upon the reference from the Ingenuity Knowledge Database. Nodes represent the molecules/genes and the relationship between two nodes is represented by edges/lines. miRNA, Micro Ribonucleic Acid; HRAS, Harvey Rat Sarcoma Viral Oncogene Homolog; CEBPB, CCAAT/Enhancer-Binding Protein (C/EBP), Beta; ARAF, A-RAF Proto-Oncogene, Serine/Threonine Kinase; PIK3CD, Phosphatidylinositol-4,5-bisphosphate 3-kinase- Catalytic Subunit Delta; CXCR4, C-X-C Chemokine Receptor 4; TGF-β1, Transforming Growth Factor Beta 1; MEF2D- Myocyte Enhancer Factor 2D; VASP, Vasodilator-Stimulated Phosphoprotein; MYL9, Myosin Light Chain 9- Regulatory; HSPG2, Heparan Sulfate Proteoglycan 2; YBX1, Y-Box Binding Protein 1.

Supplementary Figure 6. Real-time PCR of miR-663a in HK-2 cells and HK-2 cells-derived exosomes. miR-663a expression was significantly upregulated in (A) HK-2 cells under high glucose than to low glucose while significantly downregulated in HK-2 cells derived exosomes under hyperglycaemic conditions. Normality was analyzed by Graphpad Prism v7.0. The *two-tailed Student's t-test* was used to find out the significant difference between the group (n=3).

Supplementary Table

Table ST1. Raw miRNA Ct values of the HK-2 cells-derived exosomes.

Samples	UniSp6	miR-142	miR-451	miR-23a	miR-30c	miR-103a
LE1	19.11	29.78	0	27.58	27.78	28.28
LE2	19.11	32.88	36.28	29.71	30.33	30.52
LE3	19.15	29.76	26.21	26.23	26.78	27.23
HE1	19.18	29.85	26.19	26.18	26.59	27.1
HE2	19.21	31.83	26.06	28.15	28.64	29.11

HE3	19.22	31.33	26.04	27.62	28.33	28.22
Blank	19.28	0	0	40	0	0

miRNA, Micro Ribonucleic Acid; Ct, Threshold Value; HK-2, Human Kidney Proximal tubular Cell Line; LE1, LE2, LE3, Low glucose treated exosomes from three different samples; HE1, HE2, HE3, High glucose treated exosomes from three different samples; UniSp6, Spike-in cDNA synthesis control.
